# Sugar-induced modulation of biogenic amines formation and metabolic profiles during *Bacillus subtilis* fermentation

**DOI:** 10.1016/j.fochx.2025.102793

**Published:** 2025-07-16

**Authors:** Seo-Hee Kwon, Sumin Song, Hyeyoung Lee, Do Yup Lee, Min Kyung Park, Young-Suk Kim

**Affiliations:** a Department of Food Science and Biotechnology, Ewha Womans University, Seoul 03760, Republic of Korea; b Division of Applied Bioengineering, Dong-Eui University, Busan 47340, Republic of Korea; c Department of Agricultural Biotechnology, Center for Food and Bioconvergence, Research Institute for Agricultural and Life Sciences, Seoul National University, Seoul 08826, Republic of Korea; d Food Processing Research Group, Korea Food Research Institute, Wanju, 55365, Republic of Korea

**Keywords:** *Bacillus subtilis*, Biogenic amines, Metabolomics, Sugar

## Abstract

Sugars influence biogenic amines (BAs) formation during microbial fermentation, because microorganisms have distinct preferences and metabolic pathways depending on a sugar type. This study investigated the effects of sugar (glucose, fructose, or sucrose) supplementation on the formation of BAs, and volatile and non-volatile metabolic profiles in *Bacillus subtilis* fermentation. Sugar addition significantly reduced total BAs, particularly, spermidine. Glucose added sample increased acetoin production (∼9-fold), indicating activation of carbon overflow metabolism. Fructose added sample showed the accumulation of methylthioadenosine, suggesting modulation of methionine salvage. Multivariate analyses (PCA, PLS-DA, and HCA) confirmed clear group separations depending on sugar type. Metabolic pathway analysis demonstrated that a sugar type significantly affected nitrogen metabolism as well as carbon overflow pathway. These results highlighted that targeted sugar can effectively regulate BA formation as well as some volatile metabolite, such as acetoin, pyrazines, and esters, improving both safety and quality of fermented foods.

## Highlights

• The formations of biogenic amines and other metabolites by *Bacillus subtilis* can be critically affected by the type of sugars added.
• Sugar addition reduced spermidine and increased certain volatile metabolites.
• Carbon overflow reduced TCA intermediates and raised neutral metabolites.
• Sugar metabolism affected nitrogen-related pathways, including methionine, and influenced biogenic amines and pyrazines levels.

## 1 Introduction

Biogenic amines (BAs), which are hazardous nitrogen compounds, are primarily formed via microbial α-decarboxylation of amino acids catalyzed by pyridoxal phosphate-dependent bacterial decarboxylases, which remove the α-carboxyl group to generate the corresponding amines (Doeun et al., 2017). Bacterial decarboxylation is predominantly activated by two physiological factors: the activation of a defense system against oxidative stress and the production of additional energy (Gardini et al., 2016). BAs are often found in some fermented foods, requiring strict control during fermentation processing due to their heat stability and resistance to degradation as well as toxicity (Ruiz-Capillas & Herrero, 2019). Several microbial species, such as *Bacillus* spp., *Lactobacillus*, and *Enterococcus* spp., have been identified as major BA-forming bacteria in fermented foods (Burdychova & Komprda, 2007; Chang & Chang, 2012). In particular, *Bacillus subtilis,* a main microorganism in fermented foods, is known to be actively involved in the production of BAs (Postollec et al., 2011).

Sugars play a dual role in microbial fermentation: they serve as carbon sources and also influence the production of BAs and other metabolites. The types and concentrations of sugars significantly affect bacterial metabolic activities, including BAs formation. For instance, adding 0.5 %–1 % of sugars (mainly composed of glucose) reduces the levels of BAs in chorizo dry sausage fermented by lactic acid bacteria which are decarboxylase-negative strains (González-Fernández et al., 2003). Similarly, Latorre-Moratalla et al. (2010) reported that an increase in sugar content reduces cadaverine by approximately 43 % in Italian *salame abruzzese*. Some studies (Chai et al., 2012; Mao et al., 2024) have demonstrated that glucose availability modulates intracellular carbon fluxes in *B. subtilis*. Recent study has shown that dynamic regulation of glucose uptake can effectively reduce overflow metabolism and restore metabolic balance, particularly under conditions of carbon excess (Halmschlag et al., 2023; Mao et al., 2024). However, the relationship between BAs formation and volatile metabolite profiles under the addition of sugars has not be comprehensively investigated yet.

In this study, the changes in the volatile and non-volatile metabolite profiles of *B. subtilis*, a bacterium known to regulate BAs formation, were compared with the addition of different sugar types (glucose, fructose, and sucrose) to the culture medium. It aimed to elucidate the effects of sugar type on BAs formation and metabolites profile shift during *Bacillus subtilis* fermentation, with a particular focus on how sugar metabolism influences metabolic pathways related to safety and quality of fermented foods.

## 2 Material and methods

### 2.1 Chemicals and reagents

Tryptone soy broth (TSB) was purchased from Becton Dickson (Sparks, MD, USA). HPLC grade water and acetonitrile were purchased from J.T. Baker Co. (Phillipsburg, NJ, USA). All other reagents and chemicals used in the study were analytical-grade and purchased from Sigma-Aldrich (St. Louis, MO, USA).

### 2.2 Microorganism and cultivate condition

*Bacillus subtilis* (KCTC2217) was obtained from Korean Collection for Type Cultures (KCTC, Jeollabuk-do, Korea). It was inoculated (initial OD_600_ = 0.1) in TSB medium, which contained 1 % (*w*/w) of different sugars (fructose, glucose, and sucrose), and cultivated for 25 h (30 °C, 100 rpm) using a shaking incubator (Vision Scientific Co., Ltd., Daejeon, Chungcheongnam-do, Republic of Korea). After the completion of fermentation, the OD at 600 nm was measured to monitor cell growth.

### 2.3 Biogenic amines analysis

Five milliliter of cultured media was mixed with 10 mL of 0.6 M perchloric acid and 10 μL of 1,7-diaminoheptane (150 ppm), as internal standard in water. Then, samples were vortexed for 3 min and incubated for 30 min at room temperature. Samples were centrifuged (4 °C) for 10 min at 1977 xg after incubation. The supernatant was transferred into a new conical tube. For re-extraction, 10 mL of 0.6 M perchloric acid was added into the vial. Final volume was adjusted to 25 mL with 0.6 M perchloric acid. Extracted samples were diluted with acetonitrile at a ratio of 1: 10 and filtered with a membrane filter (Whatman Syringe Filter, PTFE, 0.2 μm). Finally, 2 μL of samples were injected into the LC-MS/MS.

The BA analysis was conducted using TSQ Endura mass spectrophotometer (Thermo Fisher Scientific, Maltham, MA, USA) and Ultimate 3000 high-performance liquid chromatograph (Thermo Fisher Scientific). The detection column was a HILIC column (Zorbax HILIC 100 × 2.1 mm, 3.5 μm, Agilent Technologies), at the temperature of 50 °C. For mobile phase, the gradient elution of two solvents was carried out at a flow rate was 0.2 mL/min: Solvent A consisted of 95 % 30 mM NH_4_HCO_2_ (ammonium formate) buffer solution dissolved in HPLC grade water (adjusted to pH 4.0 using formic acid) with 5 % acetonitrile, and solvent B was 95 % acetonitrile with 5 % 30 mM NH_4_HCO_2_ (ammonium formate) buffer solution dissolved in HPLC grade water (adjusted to pH 4.0 using formic acid). The gradient started at 85 % and was then decreased to 40 % in 12 min. After holding for 3 min, it was increased to 85 % acetonitrile in 0.1 min and maintained for 9.9 min. The source voltage was set to 3.5 kV for positive ionization, and the temperatures of ion transfer tube and vaporizer were 300 °C and 350 °C, respectively. The selected reaction monitoring (SRM) mode was used to obtain MS data. The quantifier ion for each compound is shown in Table S1.

The qualitative analysis was carried out using an external calibration method. For quantification, 5-point calibration curves were drawn using mixture of authentic standard compounds, diluted in HPLC-grade water and acetonitrile (Table S2). The qualitative and quantitative analysis was conducted using Xcalibur software (version 4.4, Thermo Fisher Scientific). Using external calibration, limit of detection (LOD) and limit of quantification (LOQ) were obtained for each compound, using the standard deviation of the y-intercepts and the slope of the calibration curve.

### 2.4 Volatile metabolites analysis

For the analysis of volatile metabolites, a modified method from Park et al. (2019) was employed. Eight milliliter of culture media was transferred into 10 mL glass vial (Agilent Technologies, Santa Clara, CA, USA), adding 2 μL of L-borneol (an internal standard, 100 ppm in methanol). For stir bar sorptive extraction (SBSE), Gerstel© twister for SBSE with polydimethylsiloxane (PDMS) was stirred at 1000 rpm for 60 min. Desorption was conducted with thermal desorption unit (TDU), started from 30 °C (1 min) and increased to 220 °C (5 min) at rate of 60 °C/min.

Volatile metabolites analysis was conducted using a 5977 A mass spectrometer (MS, Agilent Technologies) and HP 7890B gas chromatograph (GC) equipped with a Stabilwax column (30 m × 0.25 mm × 0.25 μm; RESTEK Corp., Bellefonte, PA, USA). The oven temperature was maintained at 40 °C (5 min), and increased to 130 °C (5 min) at a rate of 4 °C/min, followed by an increase to 220 °C (10 min) at the same rate. The mass spectrum was obtained with a scan range of 35 to 350 amu. The samples were injected in split-less mode, using helium (0.8 mL/min linear speed) as the carrier gas and the temperature of transfer line was 250 °C. The identification of metabolites was performed by comparison of retention times (RT) and mass spectra to those of authentic compounds. The volatile metabolites were identified based on their mass spectra through NIST08 and Wiley09 databases as well as retention index (RI) values. Quantitative analysis was performed by calculating their relative ratio of individual peak area compared to that of an internal standard.

### 2.5 Non-volatile metabolites analysis

For the analysis of non-volatile metabolites analysis, a modified method from Yu, Youn, et al. (2021) was applied. Methoxyamine hydrochloride (40 mg/mL pyridine) and MSTFA (*N*-methyl-*N*-trimethylsilyl trifluoroacetamide) with 1 % TMCS (trimethylchlorosilane) were used as derivatizing reagents.

For extraction, a steel bead was added in each freeze-dried samples and grinded in a mixer mill at 25 Hz for 80 s. After breaking cells, 1.2 mL of a solvent, which consists of methanol, isopropyl alcohol, water mixed in a ratio of 3:3:2 (*v*/v/v), was added. Samples with solvent were homogenized in a mixer mill at 28 Hz for 60 s and stored in ice for 60 min. Homogenized samples were centrifuged at 16,422 x*g*, 4 °C for 5 min. Then internal standards including heptanoic acid (1000 μg/mL ethanol, for organic acids and fatty acids), norleucine (1000 μg/mL water, for amino acids), and threitol (1000 μg/mL water, for sugars and sugar alcohols), were added in 1.1 mL of the supernatant and concentrated with speed vacuum concentration. For primary derivatization, 5 μL of methoxyamine hydrocholoride (40 mg/mL pyridine) was added in samples and vortexed, centrifuged, incubated in shaking incubator at 800 rpm, 30 °C for 90 min. After incubation, 2 μL of fatty acid methyl ester (FAME) was added as an internal retention time index standard. Then 45 μL of *N*-methyl-N-(trimethylsilyl)-trifluoroacetamide (MSTFA) with 1 % trimethylchlorosilane (TMCS) was added to the samples for secondary derivatization. The mixture was vortexed, centrifuged, and incubated in a shaking incubator at 800 rpm and 37 °C for 60 min.

Non-volatile metabolites analysis was carried out using Leco Pegasus HT time-of-flight mass spectrometer (LECO, St. Joseph, MI, USA) equipped with an Agilent 7890B gas chromatograph (Agilent Technologies). Each derivatized sample (0.5 μL) was injected (split-less mode) into the gas chromatograph equipped with Rtx-5Sil MS column (30 m × 0.25 mm × 0.25 μm; RESTEK Corp., Bellefonte, PA, USA). The oven temperature was maintained at 50 °C for 1 min, followed by an increase to 330 °C at a rate of 20 °C /min, and held for 5 min. The samples were injected in split-less mode, using helium (1 mL/min linear speed) as a carrier gas. The temperature of inlet and transfer line were 250 °C and 280 °C, respectively. The mass spectrum was acquired in the electron impact (EI) ionization mode (70 eV), with a scan range of 85 to 550 *m*/*z* at a rate of 20 spectra/s.

Identification of non-volatile metabolites was performed using MS-dial software. Binbase library (Fiehn lab library, University of California Davis, Davis, CA, USA) was used to process and annotate the mass spectra data of non-volatile metabolites. The RI values of non-volatile metabolites, determined using FAME, were compared to those of standard compounds. And their relative percentages of individual peak area compared to that of internal standards was estimated for quantification.

### 2.6 Statistical analysis

All experiments were conducted in triplicate. Analysis of variance (ANOVA) was performed using IBM SPSS (version 16.0, Chicago, IL, USA). To verify statistically significant differences between samples, mean values were compared using Duncan's one-way ANOVA test and multiple range test. Multivariate statistical analysis was carried out using SIMCA 16 software (Umetrics, Umea, Sweden). Principle component analysis (PCA) and partial least square-discriminant analysis (PLS-DA) were conducted with observed values of volatile and non-volatile metabolites to estimate tendencies of each sample and determine differences between samples. Hierarchical cluster analysis (HCA) was also performed using Euclidean distance and Ward's linkage method to visualize sample-level clustering patterns.

## 3 Results and discussion

Each microorganism prefers specific sugars, leading to distinctive metabolic networks depending on sugar types (Kampen, 2014; Lievens et al., 2015). In this study, two monosaccharides (glucose and fructose) and a disaccharide (sucrose) were used to investigate the effects of different sugars on the formation of BAs and volatile/non-volatile metabolites in *B. subtilis* fermentation. Glucose and fructose are reducing sugars abundant in nature and the preferred energy sources by many microorganisms. Glucose, an aldohexose, is not only the preferred carbon source of *B. subtilis*, but also the most efficiently utilized one for engineered high-yield metabolite production under redox-optimized conditions (Gu et al., 2019). Fructose, a ketohexose, is also an abundant monosaccharide (Qi & Tester, 2019). Aldohexose and ketohexose can be classified according to their functional groups, such as formyl and ketone groups, respectively. Sucrose is a widely used disaccharide in fermented foods and serves as a carbon source that can be enzymatically hydrolyzed into glucose and fructose by *B. subtilis* (Lievens et al., 2015). This allows comparative investigation on both mono and disaccharide metabolism and their combined impact on metabolic pathways. In this study, four experimental groups were prepared to investigate the effects of different sugars on the generation of BAs and metabolites: a control group without the addition of sugar (BC) and experimental groups with the addition of glucose (BG), fructose (BF), and sucrose (BS).

### 3.1 The effects of sugar addition on biogenic amines formation

Table 1 shows the contents of BAs found in each sample. Specifically, phenylethylamine (PHE), tyramine (TYR), and spermidine (SPD) were detected. The total amounts of BAs decreased in the sugar-added samples (BG, BF, and BS) compared with those in BC; however, the changes in the contents of each BA varied depending on the experimental conditions. PHE and TYR showed no significant differences among all samples, regardless of the addition of sugar and the type of sugars. Among the detected BAs, SPD was the most dominant and its content significantly decreased when sugar was added. In particular, BF had the highest BA contents, whereas BS had the lowest. The decrease in spermidine levels upon sugar addition may be attributed to altered carbon‑nitrogen metabolism. Excess glucose or fructose promotes overflow metabolism, reducing the need for amino acid decarboxylation involved in polyamine synthesis (Härtig & Jahn, 2012). The increased level of methylthioadenosine (MTA) in fructose-supplemented samples indicates a feedback effect in the methionine salvage pathway. These changes may be regulated by carbon catabolite repression, which downregulates nitrogen-related metabolism under high carbon availability (Cruz Ramos et al., 2000; Gu et al., 2019). SPD can be derived from arginine produced by urea cycle in *B. subtilis* (Sekowska et al., 1998). It participates in the regulation of gene expression and promotes biofilm formation by activating a specific gene expression *(*Burrell et al., 2010; Igarashi et al., 1982). A previous study (Aziz, 2003) suggested that SPD metabolism in plants may influence sucrose synthesis or its accumulation in related organs. A relative content of BAs, including SPD, decreased when sugars were added during the ripening period of fermented sausages compared with that when sugars were not added (Bover-Cid et al., 2000).

**Table 1:** The quantitative results of biogenic amines (BAs).

**BAs**	**Contents of BAs**^a^**(ppm)**			
	**BC**	**BF**	**BG**	**BS**
**TRP**	N.D.^b^	N.D.	N.D.	N.D.
**PHE**	0.026 ± 0.003 a^c^	0.024 ± 0.003 a	0.029 ± 0.003 a	0.026 ± 0.003 a
**PUT**	N.D.	N.D.	N.D.	N.D.
**CAD**	N.D.	N.D.	N.D.	N.D.
**HIS**	trace	trace	trace	trace
**TYR**	0.178 ± 0.048 a	0.192 ± 0.033 a	0.198 ± 0.032 a	0.178 ± 0.031 a
**SPD**	4.404 ± 0.302 c	3.555 ± 0.121 b	3.295 ± 0.013 ab	3.054 ± 0.282 a
**Total**	4.608 ± 0.312 c	3.771 ± 0.107 b	3.522 ± 0.042 ab	3.257 ± 0.309 a

TRP, tryptophan; PHE, 2-phenylethylamine; PUT, putrescine; CAD, cadaverine; HIS, histamine; TYR, tyramine; SPD, spermidine, BC, *B.subtilis* grown without additional sugars; BF, *B.subtilis* grown with addition of 1 % fructose; BG, *B.subtilis* grown with addition of 1 % glucose; BS, *B.subtilis* grown with addition of 1 % sucrose.

a Mean values of relative peak area to that of internal standard ± standard deviation.

b N.D., Not detected.

c Different letters shows significant differences (*p* < 0.05) among the samples using Duncan's multiple comparison test.

### 3.2 Metabolic changes according to sugar type

A total of 102 volatile metabolites were identified through GC–MS analysis: 3 acids, 15 alcohols, 5 aldehydes, 7 benzenes, 9 esters, 3 furans and its derivatives, 4 hydrocarbons, 16 ketones, 4 lactones, 6 phenols, 18 pyrazines, 3 sulfides, 2 ethers, and 7 N-containing compounds (**Table S3**). The volatile metabolite profiles showed that the volatile metabolites levels in the sugar-added samples increased by more than two-fold compared with those in the control. In the sugar-added samples, the total contents of acids, aldehydes, benzenes, esters, furans, hydrocarbons, ketones, lactones, and phenols increased, while the total contents of alcohols decreased. Among the detected volatile metabolites, 2,5-dimethylpyrazine and 3-hydroxybutan-2-one (acetoin) had the highest contents in all samples. The highest level of 2,5-dimethylpyrazine was detected in BC (constituting 18.73 % of total amounts), while the highest level of acetoin was observed in sugar-added samples, including BS, BG, and BF (approximately 60 % of total amounts).

**Fig. 1:**
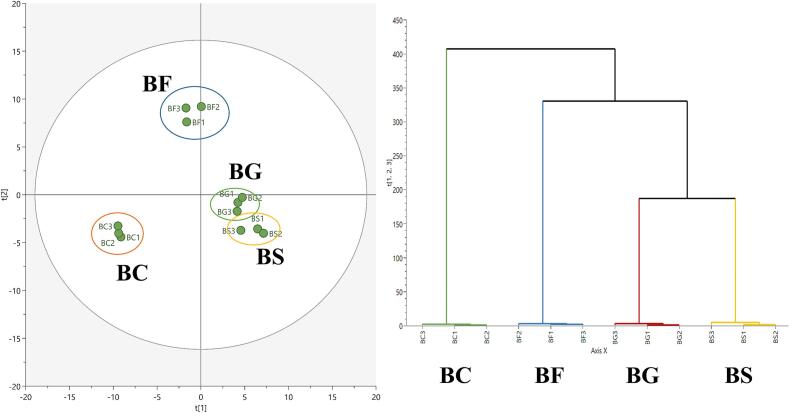
PCA score plot and HCA based on the volatile metabolites in *Bacillus subtilis*. Each node indicates as follow: BC, control (Green); BF, 1 % fructose added samples (Blue); BG, 1 % glucose added samples (Red); BS, 1 % sucrose added samples. (For interpretation of the references to color in this figure legend, the reader is referred to the web version of this article.)

Sugar is an important energy source for bacterial growth and precursor for various metabolic compounds; as such, changes in volatile and non-volatile metabolites were investigated when different sugar types were added. Principal component analysis (PCA) was conducted to distinguish the metabolic differences among the samples. Fig. 1 shows the PCA score plot based on the volatile metabolite profiles. The PCA model explained 83 % of the total variance, and the model parameters were R^2^X = 0.834 and Q^2^ = 0.661. In addition, hierarchical cluster analysis (HCA) was conducted to further validate the inter-group metabolic differences. In the score plot, BC was in a dimension that differed from that of BS, BG, and BF. This result demonstrated that relatively obvious metabolic changes occurred when sugars were added to the cultivated media. Conversely, BS and BG were closer to each other than to other sample groups and located in the same dimension. It could suggest that the metabolic profile of BS was more influenced by glucose, likely due to the preferential utilization of glucose over fructose following sucrose hydrolysis by *B. subtilis*. This finding suggested that sugar addition to BC caused metabolic changes in BS and BG that were more similar to each other than to BF. *B. subtilis* possesses a digestive enzyme that catalyzes the hydrolysis of sucrose to fructose and glucose; it prefers glucose to fructose as the carbon source. Gu et al. (2019) reported that glucose is the preferred carbon source of *B.subtilis*. Therefore, this study focused on the metabolic changes in BG and BF in detail and excluded BS.

**Fig. 2:**
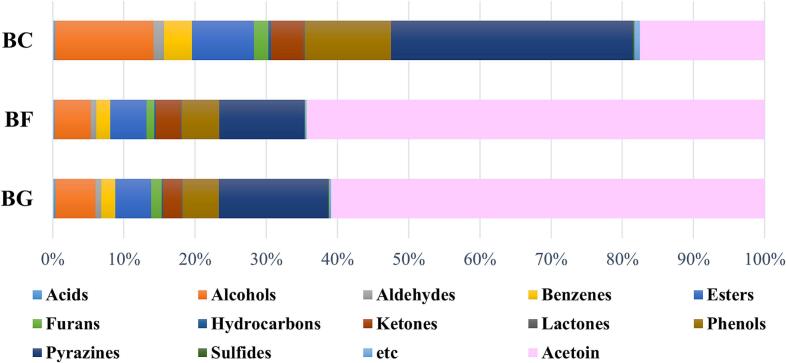
Volatile metabolites (excluding acetoin) grouped by chemical classes. (A) Total metabolites. Acetoin was excluded in grouping, instead noted additionally. (B) Main groups detected in high levels. Expressed with relative peak area compared to that of internal standard. Asterisk indicates a statistically significant difference (*p*-value <0.05) in the changes of chemical groups compared to BC.

Fig. 2**.(A)** shows the percentages of volatile metabolite contents classified by chemical functional groups. Acetoin was separately indicated because it accounted for up to 64 % of the total volatile metabolites. In general, pyrazines, alcohols, phenols, and ketones show high composition ratios in the total volatile metabolites. Pyrazines are mainly formed through non-enzymatic reactions between reducing sugars and amino acids in foods (Yu, Zhang, et al., 2021). However, *B.subtilis* can produce pyrazines through enzymatic activities involving threonine and acetoin (Kłosowski et al., 2021). In this study, when sugar was added, the proportion of pyrazines in the total volatile metabolites decreased in BG and BF compared with that in BC (Fig. 2(B)). However, it increased quantitatively compared with that in the control when glucose was added, it decreased in BF. The contents of 2-(3-methylbutyl)pyrazine, 2,5-dimethyl-3-(3-methylbutyl)pyrazine, 1-(6-methylpyrazin-2-yl)ethanone, and 2-ethyl-6-methylpyrazine also increased when sugars were added. In addition, 2,5-dimethyl-3-(2-methylpropyl)pyrazine was detected only in sugar-added samples. However, 2,6-diethylpyrazine and 1-(5-methylpyrazin-2-yl)ethenone were found only in BF. These results might be associated with the quantitative changes in acetoin. Acetoin, which is used as a food flavor additive in dairy products, is one of the precursors in pyrazine production (Zhang et al., 2019; Zhu et al., 2010). The composition ratio and content of acetoin significantly increased in the sugar-added samples compared with those in BC. Previous studies (Gu et al., 2019; Xu et al., 2018) showed that sugar addition increases the contents of some metabolites, including acetoin, ethanol, and acetic acid, via overflow pathways for excess carbons and reductants. *B.subtilis,* which is cultivated under aerobic condition, excretes acetic acid to reproduce NAD^+^ consumed by glycolysis or to recover coenzyme A (CoASH) for the conversion of pyruvate to acetyl-CoA (Wolfe, 2005). Ethanol excretion via the conversion from acetyl-CoA contributes to NADH reoxidation for replenishing NAD^+^ (Nakano et al., 1997). However, acetic acid production results in the acidification of cellular internal and external pH. Accumulated pyruvate is converted to pH-neutral metabolites, such as acetoin and 2,3-butanediol (Härtig & Jahn, 2012), to maintain pH balance. In this study, acetates, including ethyl acetate, butyl acetate, 3-methylbutyl acetate, ethyl 2-phenylacetate, 2-phenylethyl acetate, and ethyl2-hydroxy-2-phenylacetate, were composed of approximately 40 % of total esters, and their contents increased in the sugar-added samples. Acetic acid is easily found as a byproduct of the overflow mechanism when glucose or other preferred carbon sources are supplied in the media (Cruz Ramos et al., 2000; Presecan-Siedel et al., 1999). In this study, acetic acid was detected only in the sugar-added samples; conversely, ethanol was not found in BF, but it increased in BG compared with that in BC. Wilks et al. (2009) reported that acid stress stimulates alcohol dehydrogenase production, which contributes to ethanol synthesis. In summary, acidification caused by an excessive carbon source supply might enhance the formation of ethanol, acetate, and acetoin in the sugar-added samples.

The generation of furans was distinctive in the changes in the volatile metabolite profiles when sugar was added. 5-(hydroxymethyl)Furan-2-carbaldehyde (5-HMF), which is commonly used as a flavoring and coloring additive (Abraham et al., 2011), was found in all the samples, while furan-2-carbaldehyde (furfural) and furan-2-ylmethanol (furfuryl alcohol) were detected only in the sugar-added samples. 5-HMF is formed through acid catalyzed carbohydrate dehydration (De Souza et al., 2012). Furfural is derived from the metabolism of a polysaccharide composed of pentose, which is also catalyzed by acids. Furfural can act as an exclusive precursor in the synthesis of molecules containing furyl, furfuryl, furoyl, or furfurylidine radical (Hoydonckx et al., 2000). Becerra et al. (2022) demonstrated that furfuryl alcohol, which is derived from furfural, is produced through reductive furfural degradation by *B.subtilis*.

In this study, a total of 221 non-volatile metabolites were identified through GC-TOF/MS: 41 amino acids, 21 fatty acids, 34 organic acids, 8 sugar acids, 11 sugar alcohols, 26 sugars, and 80 others (**Table S4**). The total contents of amino acids, organic acids, and sugar acids decreased when sugars were added, although some individual metabolites within each group showed increased levels. In particular, the total amino acid contents decreased by 4.5- and 8.5-fold in BG and BF, respectively, compared with those in BC. The organic acid contents and sugar acids decreased by 6-fold to 7-fold and 1.3-fold to 1.8-fold, respectively, in the sugar-added samples compared with those in BC.

**Fig. 3:**
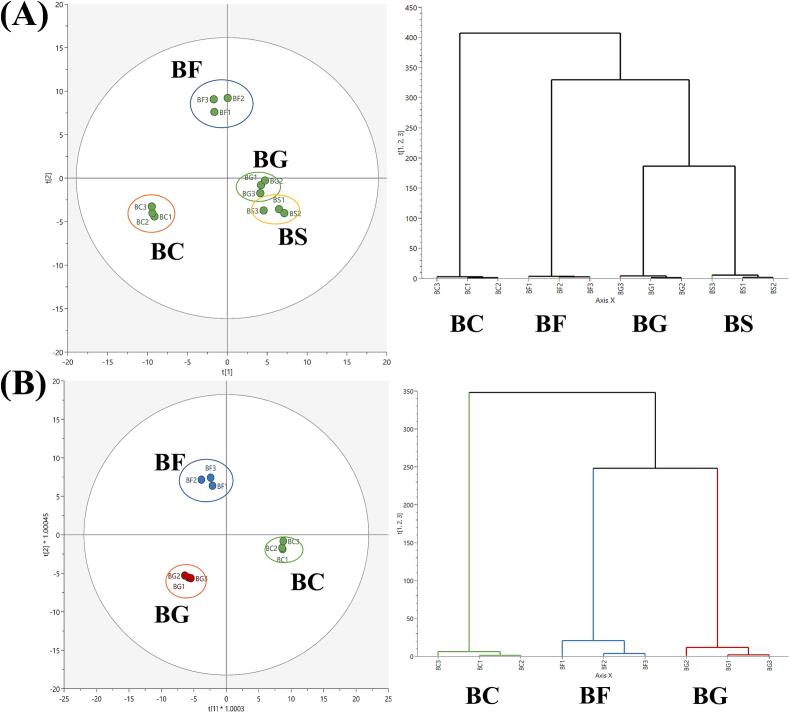
PCA score plot and HCA (A) and PLS-DA score plot and HCA (B) based on the non-volatile metabolites in *Bacillus subtilis*. Each node indicates as follow: Green, control (BC); Blue, 1 % fructose added samples (BF); Red, 1 % glucose added samples (BG). (For interpretation of the references to color in this figure legend, the reader is referred to the web version of this article.)

Fig. 3 shows the score plots of PCA and PLS-DA based on the non-volatile metabolite profiles from BC, BG, and BF. Since sucrose was hydrolyzed into glucose and fructose by *B. subtilis*, resulting in a metabolic profile largely overlapping with BG (Fig. 1), we focused detailed analysis on BC, BF, and BG to highlight clearer distinctions. The PCA model explained 73.5 % of the total variance, and the model parameters were R^2^X = 0.735 and Q^2^ = 0.635. The other model parameters were R^2^X = 0.732, R^2^Y = 0.991, and Q^2^ = 0.939. The suitability of this model was determined by obtaining R^2^ and Q^2^ (R^2^ = 0.492 and Q^2^ = −0.255, Fig. S1) through a permutation test. Variables with absolute values of p(corr) > 0.8 and VIP > 1.3 were considered the main metabolites that could be distinguished various samples (Saccenti et al., 2014; Xu et al., 2024). Table 2 lists the main non-volatile metabolites contributing to discrimination of different samples. Simple sugars and sugar alcohols, such as galactose, isohexonic acid, glucose, erythritol, mannitol and arabinose, were major metabolites contributing to the negative axis of PC2 (sugar-added samples).

**Table 2:** Main non-volatile metabolites using PLS-DA model.

**Main non-volatile metabolites**^a^	**VIP**^b^	**pcorr**^c^
***Control-contributing***		
Melibiose	1.317	0.998
Valine	1.316	0.994
Adenine	1.316	0.993
glutamic acid	1.316	0.995
Proline	1.315	0.994
β-glycerolphosphate	1.315	0.992
glycerol-α-phosphate	1.315	0.993
UDP-*N*-acetylglucosamine	1.314	0.993
phosphogluconic acid	1.313	−0.991
Pinitol	1.312	0.993
β-sitosterol	1.309	0.989
Cholesterol	1.308	0.988
fumaric acid	1.307	0.992
nicotinic acid	1.307	0.986
citraconic acid	1.307	0.991
Ononitol	1.307	0.988
Maltitol	1.305	0.987
succinic acid	1.305	0.987
Melezitose	1.304	0.986
Raffinose	1.304	0.986
Hexitol	1.304	0.986
methionine sulfoxide	1.303	0.985
1-kestose	1.303	0.986
Galactitol	1.301	0.985


**Main non-volatile metabolites**^a^	**VIP**^b^	**pcorr**^c^
***Samples with sugar-contributing***		
isohexonic acid	1.649	−0.880
*N*-methylalanine	1.540	0.845
5′-deoxy-5′-methylthioadenosine (Methylthioadenosine, MTA)	1.563	0.835
2-hydroxybutanoic acid	1.700	0.931
Mannitol	1.440	−0.826
Glucose	1.577	−0.911
Erythritol	1.568	−0.896
Arabinose	1.418	−0.831
Galactose	1.691	−0.971

a Variables with absolute values of p(corr) > 0.8 and VIP > 1.3, listed in the order of VIP.

b The projection values of variable importance (VIP).

c The correlation coefficient values of variable importance (pcorr).

**Fig. 4:**
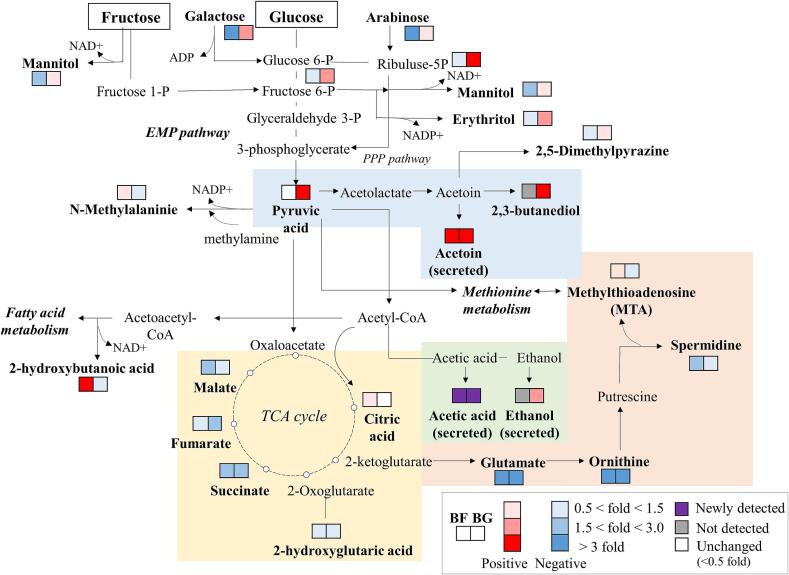
The possible metabolic pathways of specific metabolites in *B.subtilis* fermentation cultivated with addition of different sugars. The fold changes represent each relative volatile and non-volatile metabolite level of BF and BG compared to BC. Each color indicates as follows: up-expressed metabolites are represented by a red color, while blue down-expressed metabolites are shown in a blue color. Abbreviations are as follows: BC, *B.subtilis* grown without additional sugar; BF, *B.subtilis* grown with an addition of 1 % fructose; BG, *B.subtilis* grown with an addition of 1 % glucose. (For interpretation of the references to color in this figure legend, the reader is referred to the web version of this article.)

Fig. 4 illustrates the changes in the volatile and non-volatile metabolites derived from the addition of different sugars via the possible metabolic pathways of *B. subtilis* based on the KEGG pathway and a previous study (Sekowska et al., 2004). The fold changes in the volatile and non-volatile metabolite contents were calculated by comparing them with those of BC. The increased metabolites are indicated in red, while the decreased metabolites are shown in a blue box. The results depicted the changes in the main non-volatile metabolites, which were derived from the PLS-DA model, and the main volatile metabolites, which were involved in carbohydrate metabolism, such as EMP pathway, PPP pathway, and TCA cycle. Carbon sources, such as glucose and fructose, which are the primary energy sources of *Bacillus*, can considerably alter metabolic pathways. These changes likely indicate how *Bacillus* metabolizes carbon sources; for example, such changes include how *Bacillus* produces energy and forms bioformation. Pyruvates become to other metabolites with two to three carbons rather than entering the citric acid cycle (Gu et al., 2019). The TCA cycle-related metabolites, such as malate, fumarate, and succinate, decreased in the sugar-added samples. Conversely, overflow pathways-related metabolites that form acetoin, 2,3-butanediol, and acetic acid, increased compared with those in BC. The increase in these metabolites could be associated with bacteria activity in maintaining pH homeostasis, in the presence of an excessive carbon supply (Härtig & Jahn, 2012). When fructose was added to the medium, the generation of sugar alcohols, such as mannitol and erythritol, decreased; conversely, when glucose was added, the formation of these sugar alcohols increased. It might be explained by the increase in sugars and sugar alcohols associated with the EMP pathway, indicating that *Bacillus* prefers glucose as a carbon source. In this study, sugar metabolism was linked to nitrogen source metabolism. These findings indicate that sugar-induced carbon flow reduced the need for amino acid decarboxylation, thereby suppressing BAs formation. In particular, key precursors, such as ornithine and methionine, were excluded from spermidine synthesis under high carbon availability. Among pyrazines generated via *Bacillus* fermentation, 2,5-dimethylpyrazine showed the highest content, regardless of samples, showing statistically significant differences between those added with different sugar types. 2,5-Dimethylpyrazine has a characteristic roasted peanut flavor, which can remarkably influence the quality of diverse fermented foods (Baker et al., 2003). Its content significantly increased when glucose was added, but it decreased when fructose was added. The overall content of pyrazines showed a similar trend.

When fructose was added as a supplementary carbon source, the metabolites other than those related to sugar metabolism changed. BAs, including putrescine and spermidine, have precursors, such as glutamate or ornithine. In this study, the spermidine content decreased regardless of sugar type, and its related precursors decreased. Methionine metabolism is involved in the transformation of putrescine to spermidine; in this reaction, the intermediate metabolite, methylthioadenosine (Sekowska et al., 2004), increased when fructose was added. The spermidine content significantly decreased when fructose was added compared with that when glucose was added. In this study, the overall metabolic process was complex and interconnected. It also involved directly and indirectly related metabolic pathways.

## 4 Conclusion

This study investigated the effects of sugar supplementation on the formations of BAs, and volatile and non-volatile metabolites during *B.subtilis* fermentation. Our results revealed that total BAs levels decreased by the addition of sugars. Also, metabolic profiling showed that sugar supplementation affected both volatile and non-volatile metabolites relevant to the quality of foods. The addition of sugars activated the carbon overflow metabolism, characterized by reduced levels of TCA cycle intermediates and increased conversion of pyruvic acid into pH-neutral compounds, along with enhanced NAD^+^ reproduction. Some volatile metabolites, such as acetic acid, furfural, and furfuryl alcohol, were significantly generated under sugar-supplemented conditions. In addition, sugar metabolism was linked to alterations in nitrogen metabolism, including changes in methionine pathways and the formation of pyrazines and BAs. Pyrazines and acetoin shared their precursors, amino acids, and the related formation pathways with spermidine. In particular, specific carbon fluxes induced volatile metabolite production, while simultaneously suppressing biogenic amine synthesis. The fermentation conditions were fixed to focus on the effects of type of sugar in this study. Further research is needed to optimize these conditions to enhance metabolite production and control BA formation. While previous studies (Gu et al., 2019; Halmschlag et al., 2023) have mainly focused on specific sugar supplementation or targeted pathway, for example a glucose system, this study compared various sugar types under identical conditions and integrates both volatile and non-volatile metabolic data, offering a more comprehensive metabolic insight in *B. subtilis* fermentation. This metabolic regulation strategy may offer an effective tool to improve both safety and quality of fermented foods. In particular, these results suggest that specific sugars can be preferentially utilized or channeled through more efficient metabolic pathways in *B. subtilis*, influencing both BA regulation and the overall quality of fermentation.

## CRediT authorship contribution statement

**Seo-Hee Kwon:** Writing – original draft, Methodology, Formal analysis, Data curation. **Sumin Song:** Methodology, Formal analysis. **Hyeyoung Lee:** Methodology, Formal analysis, Data curation. **Do Yup Lee:** Methodology, Formal analysis, Data curation. **Min Kyung Park:** Writing – review & editing, Formal analysis, Data curation, Conceptualization. **Young-Suk Kim:** Writing – review & editing, Supervision, Conceptualization.

## Declaration of competing interest

The authors declare that they have no known competing financial interests or personal relationships that could have appeared to influence the work reported in this paper.

## Data Availability

No data was used for the research described in the article.

## Appendix A Supplementary data

Supplementary material Table S1. The quantifier ion for each biogenic amines. Table S2. The results of the validation of biogenic amines (BAs) analysis using LC-MS/MS. Table S3. Volatile metabolites identified in the samples cultivated by *B. subtilis* Table S4. Non-volatile metabolites identified in the samples cultivated by *B. subtilis*. Supplementary material
