# Supplementary material for: Sugar-induced modulation of biogenic amines formation and metabolic profiles during Bacillus subtilis fermentation
Source: Food Chem X. 2025 Jul 16;29:102793. doi: 10.1016/j.fochx.2025.102793 (PMC12296466; doi:10.1016/j.fochx.2025.102793)
Supplement: Supplementary material — Table S1. The quantifier ion for each biogenic amines. Table S2. The results of the validation of biogenic amines (BAs) analysis using LC-MS/MS. Table S3. Volatile metabolites identified in the samples cultivated by B. subtilis Table S4. Non-volatile metabolites identified in the samples cultivated by B. subtilis. [file mmc1.docx]

**Supplemental materials**

**Sugar-induced modulation of biogenic amines formation and metabolic profiles during *Bacillus subtilis* fermentation**

Seo-Hee Kwon, Sumin Song, Hyeyoung Lee, Do-Yup Lee, Min-Kyung Park*, and Young-Suk Kim*

Table S1. The quantifier ion for each biogenic amines.

| **Compound^a^** | **Parent ion (m/z)** | **Product ion (m/z)** | **CE^b^** | **RT (min)^c^** |
| --- | --- | --- | --- | --- |
| **PUT** | 72 | 30.1 | 23 | 8.99 |
| **CAD** | 86.1 | 69 | 21 | 8.66 |
| **HIS** | 112.2 | 95 | 21 | 6.16 |
| **PHE** | 122.1 | 105 | 28 | 2.73 |
| **DAHP** | 131 | 114.1 | 17 | 7.93 |
| **TYR** | 138.2 | 121 | 28 | 3.50 |
| **SPD** | 146 | 72 | 15 | 10.95 |
| **TRP** | 161.2 | 144 | 32 | 3.03 |

^a^ PUT, putrescine; CAD, cadaverine; HIS, histamine; PHE, 2-phenylethylamine; DAHP, 1,7-diaminoheptane; TYR, tyramine; SPD, spermidine; TRP, tryptophan

^b^ CE: collision energy

^c^ RT: retention time

Table S2. The results of the validation of biogenic amines (BAs) analysis using LC-MS/MS.

| **Compound** | **RT (min)** | **Range (ppm)** | **R^2^** | **LOD (ppm)** | **LOQ (ppm)** |
| --- | --- | --- | --- | --- | --- |
| **PHE** | 2.73 | 0.0010 - 1.0000 | 0.9999 | 0.00003 | 0.00011 |
| **TYR** | 3.5 | 0.0010 - 1.0000 | 0.9999 | 0.00021 | 0.00064 |
| **HIS** | 6.16 | 0.0010 - 1.0000 | 0.9999 | 0.00003 | 0.00010 |
| **SPD** | 10.95 | 0.0010 – 1.0000 | 0.9998 | 0.000329 | 0.00998 |

PHE, 2-phenylethylamine; TYR, tyramine; HIS, histamine; SPD, spermidine, RT: retention time; Conc. Range: concentration range; R^2^: square of regression coefficient; LOD: detection limit; LOQ: quantification limit

Table S3. Volatile metabolites identified in the samples cultivated by *B. subtilis*

| **No.^a^** | **Volatile metabolites** | **RI^b^** | **Relative peak area^c^ (Mean ± SD)** | | | | **ID^d^** |
| --- | --- | --- | --- | --- | --- | --- | --- |
|  |  |  | **BC** | **BF** | **BG** | **BS** |  |
|  | ***Alcohols*** |  |  |  |  |  |  |
| a1 | Ethanol | 605 | 0.051±0.005 b^e^ | N.D.^f^ a | 0.160±0.014 c | 0.149±0.017 c | B |
| a2 | butan-1-ol | 1148 | 0.251±0.011 c | 0.144±0.007 a | 0.167±0.008 b | 0.160±0.007 ab | A |
| a3 | 3-methylbutan-1-ol | 1206 | 0.063±0.005 a | 0.128±0.004 b | 0.148±0.004 c | 0.161±0.004 d | B |
| a4 | octan-2-ol | 1419 | 0.006±0.000 b | N.D. a | 0.008±0.001 c | N.D. a | B |
| a5 | oct-1-en-3-ol | 1451 | 0.032±0.001 c | 0.014±0.001 a | 0.023±0.001 b | 0.023±0.001 b | A |
| a6 | 2-ethylhexan-1-ol | 1490 | 0.625±0.033 b | 0.714±0.024 c | 0.664±0.035 b | 0.516±0.006 a | A |
| a7 | octan-1-ol | 1557 | N.D.^e^ a | 0.026±0.002 b | 0.027±0.003 b | 0.030±0.003 b | A |
| a8 | furan-2-ylmethanol | 1661 | N.D. a | 0.020±0.003 a | 0.113±0.028 b | 0.118±0.020 b | A |
| a9 | 2-(2-butoxyethoxy)ethanol | 1786 | 0.084±0.002 c | 0.089±0.008 c | 0.057±0.010 b | 0.038±0.002 a | B |
| a10 | (2E)-3,7-dimethylocta-2,6-dien-1-ol | 1848 | 0.078±0.003 a | 0.109±0.006 c | 0.103±0.002 c | 0.091±0.002 b | A |
| a11 | 2-phenylethanol | 1908 | 0.138±0.005 a | 0.197±0.007 c | 0.153±0.002 b | 0.137±0.001 a | A |
| a12 | dodecan-1-ol | 1969 | N.D. a | 0.064±0.007 b | N.D. a | N.D. a | A |
| a13 | (6E)-3,7,11-trimethyldodeca-1,6,10-trien-3-ol | 2041 | 0.219±0.009 c | N.D. a | 0.070±0.010 b | 0.073±0.003 b | A |
| a14 | tetradecan-1-ol | 2173 | N.D. a | N.D. a | 0.016±0.007 b | 0.018±0.006 b | A |
| a15 | 2-(2-dodecoxyethoxy)ethanol | >2600 | 0.043±0.007 a | 0.062±0.005 a | 0.063±0.007 a | 0.039±0.024 a | B |

Table S3. cont.

|  | ***Benzenes*** |  |  |  |  |  |  |
| --- | --- | --- | --- | --- | --- | --- | --- |
| b1 | toluene | 1040 | 0.022±0.005 a | 0.021±0.004 a | 0.028±0.001 a | 0.030±0.004 a | A |
| b2 | pyridine | 1177 | 0.011±0.002 a | 0.015±0.002 a | 0.016±0.003 a | 0.013±0.002 a | B |
| b3 | benzene-1,4-diamine | 1328 | N.D. a | N.D. a | N.D. a | 0.045±0.006 b | C |
| b4 | 4-methylquinazoline | 2070 | 0.094±0.003 ab | 0.087±0.006 a | 0.097±0.005 b | 0.095±0.002 b | C |
| b5 | 4-methylquinoline | 2091 | 0.071±0.003 a | 0.069±0.003 a | 0.076±0.003 a | 0.074±0.003 a | A |
| b6 | 3-hydroxy-4-phenylbutan-2-one | 2257 | 0.097±0.006 a | 0.228±0.033 b | 0.182±0.037 b | 0.185±0.003 b | B |
| b7 | 1-ethynyl-3-(trifluoromethyl)benzene | 2302 | 0.242±0.011 a | 0.253±0.010 ab | 0.270±0.012 b | 0.268±0.010 b | C |
|  | ***Acids*** |  |  |  |  |  |  |
| c1 | acetic acid | 1461 | N.D. a | 0.046±0.010 b | 0.049±0.014 b | 0.056±0.007 b | A |
| c2 | acetamide | 1756 | N.D. a | N.D. a | 0.038±0.013 b | 0.045±0.021 b | B |
| c3 | hexadecanoic acid | >2600 | 0.042±0.001 a | 0.044±0.015 a | 0.043±0.010 a | 0.062±0.003 a | A |
|  | ***Aldehydes*** |  |  |  |  |  |  |
| d1 | hexanal | 1090 | 0.018±0.007 a | 0.036±0.012 a | 0.033±0.006 a | 0.024±0.010 a | B |
| d2 | nonanal | 1392 | N.D. a | 0.025±0.004 b | 0.078±0.009 d | 0.053±0.011 c | B |
| d3 | decanal | 1497 | 0.127±0.007 a | 0.154±0.012 b | 0.149±0.017 ab | 0.162±0.003 b | A |
| d4 | benzaldehyde | 1523 | 0.022±0.000 a | 0.027±0.004 a | 0.027±0.003 a | 0.025±0.002 a | A |
| d5 | dodecanal | 1707 | N.D. a | N.D. a | 0.019±0.004 b | N.D. a | A |
|  | ***Ethers*** |  |  |  |  |  |  |
| et1 | 2-hexadecoxyethanol | 2240 | N.D. a | 0.018±0.002 b | N.D. a | N.D. a | C |
| et2 | 2-dodecoxyethanol | 2241 | 0.019±0.001 bc | N.D. a | 0.025±0.005 c | 0.018±0.003 b | C |

Table S3. cont.

|  | ***Esters*** |  |  |  |  |  |  |
| --- | --- | --- | --- | --- | --- | --- | --- |
| e1 | ethyl acetate | 885 | N.D. a | N.D. a | 0.042±0.002 c | 0.032±0.004 b | B |
| e2 | ethyl 2-methylbutanoate | 1054 | N.D. a | N.D. a | 0.016±0.002 b | 0.017±0.002 b | A |
| e3 | butyl acetate | 1077 | 0.090±0.016 a | 0.085±0.008 a | 0.127±0.003 b | 0.089±0.006 a | A |
| e4 | 3-methylbutyl acetate | 1127 | N.D. a | 0.031±0.008 bc | 0.044±0.013 c | 0.022±0.002 b | A |
| e5 | butyl 2-methylprop-2-enoate | 1227 | N.D. a | N.D. a | 0.012±0.007 b | N.D. a | C |
| e6 | ethyl 2-phenylacetate | 1782 | 0.023±0.001 a | 0.052±0.003 b | 0.179±0.010 c | 0.285±0.001 d | A |
| e7 | 2-phenylethyl acetate | 1813 | 0.028±0.004 a | 0.046±0.004 b | 0.047±0.011 b | 0.035±0.006 ab | A |
| e8 | 2-phenylethyl 3-methylbutanoate | 1984 | N.D. a | 0.018±0.004 b | N.D. a | N.D. a | A |
| e9 | ethyl 2-hydroxy-2-phenylacetate | >2600 | 0.102±0.009 a | 0.118±0.007 ab | 0.130±0.010 b | 0.159±0.007 c | B |
|  |  |  |  |  |  |  |  |

Table S3. cont.

|  | ***Furan and furan derivatives*** |  |  |  |  |  |  |
| --- | --- | --- | --- | --- | --- | --- | --- |
| f1 | furan-2-carbaldehyde | 1467 | N.D. a | N.D. a | N.D. a | 0.019±0.002 b | A |
| f2 | furan-2-ylmethanol | 1661 | N.D. a | 0.020±0.003 a | 0.113±0.028 b | 0.118±0.020 b | A |
| f3 | 5-(hydroxymethyl)furan-2-carbaldehyde | 2509 | 0.038±0.006 a | 0.046±0.003 ab | 0.068±0.020 b | 0.063±0.005 b | A |
|  | ***Hydrocarbons*** |  |  |  |  |  |  |
| h1 | hexadecane | 1597 | 0.029±0.004 a | 0.038±0.012 a | 0.030±0.008 a | 0.034±0.003 a | A |
| h2 | octadecane | 1796 | 0.024±0.002 ab | 0.028±0.004 ab | 0.028±0.012 a | 0.031±0.003 b | A |
| h3 | icosane | 1997 | N.D. a | N.D. a | N.D. a | 0.013±0.000 b | A |
| h4 | cyclotetradecane | 2591 | N.D. a | N.D. a | N.D. a | 0.034±0.003 b | A |
|  | ***Ketones*** |  |  |  |  |  |  |
| k1 | propan-2-one | 810 | 0.088±0.005 a | 0.131±0.019 b | 0.175±0.002 c | 0.150±0.012 b | B |
| k2 | heptan-2-one | 1182 | 0.040±0.003 a | 0.041±0.008 a | 0.044±0.006 a | 0.035±0.001 a | B |
| k3 | 6-methylheptan-2-one | 1236 | 0.017±0.001 a | 0.019±0.001 a | 0.020±0.002 a | 0.019±0.001 a | A |
| k4 | 5-methylheptan-2-one | 1254 | 0.061±0.003 a | 0.080±0.007 b | 0.080±0.008 b | 0.080±0.002 b | B |
| k5 | 3-hydroxybutan-2-one | 1282 | 2.40±0.102 a | 21.9±0.853 c | 20.4±0.832 b | 18.7±1.06 b | A |
| k6 | 6-methylhept-5-en-2-one | 1337 | N.D. a | N.D. a | N.D. a | 0.010±0.001 b | B |
| k7 | [1,2,4]triazolo[4,3-a]pyridin-3-amine | 1567 | N.D. a | 0.012±0.001 b | N.D. a | N.D. a | C |
| k8 | 1-(1,3-thiazol-2-yl)ethanone | 1644 | N.D. a | 0.045±0.011 b | 0.047±0.005 bc | 0.063±0.004 c | A |
| k9 | 1-phenylethanone | 1647 | 0.116±0.009 b | 0.088±0.002 a | 0.118±0.006 b | 0.129±0.006 b | B |
| k10 | 1-phenylpropan-2-one | 1722 | 0.053±0.004 b | 0.042±0.004 a | 0.046±0.003 ab | 0.050±0.003 b | A |
| k11 | 1-phenylbutan-1-one | 1790 | N.D. a | 0.030±0.005 b | 0.025±0.002 b | 0.033±0.005 b | A |
| k12 | (1S,5R)-6,8-dioxabicyclo[3.2.1]oct-2-en-4-one | 1980 | N.D. a | N.D. a | N.D. a | 0.041±0.004 b | C |

Table S3. cont.

| k13 | 1-(2-aminophenyl)ethanone | 2214 | 0.084±0.004 b | 0.078±0.004 ab | 0.073±0.005 a | 0.083±0.003 b | A |
| --- | --- | --- | --- | --- | --- | --- | --- |
| k14 | (2-methoxyphenyl)-(4-methylphenyl)methanone | 2425 | N.D. a | 0.020±0.004 b | N.D. a | N.D. a | C |
| k15 | 7,9-ditert-butyl-1-oxaspiro[4.5]deca-6,9-diene-2,8-dione | >2600 | 0.031±0.005 a | 0.296±0.017 c | 0.061±0.005 b | 0.048±0.001 ab | C |
| k16 | 1,3-diphenylpropan-1-one | >2600 | N.D. a | 0.026±0.003 b | 0.036±0.002 c | 0.032±0.002 c | C |
|  | ***Lactones*** |  |  |  |  |  |  |
| l1 | 2H-furan-5-one | 1549 | N.D. a | 0.013±0.001 ab | 0.029±0.009 bc | 0.034±0.011 c | B |
| l2 | 5-pentyloxolan-2-one | 2022 | 0.012±0.001 b | N.D. a | N.D. a | 0.014±0.001 c | A |
| l3 | 5-hexyloxolan-2-one | 2138 | 0.018±0.002 a | 0.021±0.002 a | 0.025±0.004 b | 0.028±0.001 b | B |
| l4 | (5S)-5-(hydroxymethyl)oxolan-2-one | 2484 | N.D. a | N.D. a | N.D. a | 0.024±0.009 c | C |
|  | ***Phenols*** |  |  |  |  |  |  |
| ph1 | 2-methoxyphenol | 1860 | 0.213±0.012 a | 0.265±0.014 b | 0.290±0.014 c | 0.294±0.011 c | B |
| ph2 | phenol | 2008 | 0.038±0.000 a | 0.110±0.003 b | 0.140±0.006 d | 0.127±0.007 c | A |
| ph3 | 4-ethenyl-2-methoxyphenol | 2196 | 0.783±0.025 c | 0.702±0.045 b | 0.581±0.015 a | 0.574±0.015 a | B |
| ph4 | 2,6-dimethoxyphenol | 2267 | 0.041±0.002 a | 0.259±0.013 b | 0.252±0.017 b | 0.250±0.005 b | A |
| ph5 | 4-ethenylphenol | 2401 | 0.375±0.017 b | 0.322±0.016 a | 0.340±0.013 a | 0.314±0.009 a | B |
| ph6 | 4-(2-methylbutan-2-yl)phenol | 2414 | 0.024±0.002 a | 0.034±0.001 b | 0.026±0.002 a | 0.025±0.002 a | C |

Table S3. cont.

|  | ***Pyrazines*** |  |  |  |  |  |  |
| --- | --- | --- | --- | --- | --- | --- | --- |
| p1 | pyrazine | 1208 | 0.070±0.002 c | 0.042±0.005 b | 0.048±0.009 b | N.D. a | A |
| p2 | 2-methylpyrazine | 1260 | 0.208±0.005 c | 0.163±0.006 a | 0.199±0.009 b | 0.185±0.009 b | A |
| p3 | 2,5-dimethylpyrazine | 1315 | 2.65±0.086 b | 2.06±0.061 a | 3.02±0.035 c | 2.89±0.100 c | A |
| p4 | 2,6-dimethylpyrazine | 1322 | 0.313±0.052 a | 0.337±0.021 a | 0.323±0.004 a | 0.380±0.071 a | A |
| p5 | 2-ethyl-6-methylpyrazine | 1379 | 0.025±0.001 a | 0.028±0.001 ab | 0.030±0.003 b | 0.037±0.004 c | B |
| p6 | 2,3,5-trimethylpyrazine | 1396 | 0.277±0.010 c | 0.236±0.006 a | 0.255±0.005 b | 0.249±0.009 ab | A |
| p7 | 2-methyl-5-propan-2-ylpyrazine | 1405 | 0.081±0.003 b | 0.073±0.001 a | 0.102±0.003 c | 0.104±0.000 c | B |
| p8 | 3-ethyl-2,5-dimethylpyrazine | 1440 | 0.302±0.012 ab | 0.285±0.010 a | 0.319±0.005 b | 0.311±0.009 b | B |
| p9 | 2,6-diethylpyrazine | 1456 | N.D. a | 0.024±0.001 b | N.D. a | N.D. a | B |
| p10 | 2-ethenyl-6-methylpyrazine | 1485 | 0.025±0.001 c | 0.023±0.001 bc | 0.020±0.001 a | 0.021±0.001 ab | B |
| p11 | 2,5-dimethyl-3-(2-methylpropyl)pyrazine | 1520 | N.D. a | 0.020±0.004 b | 0.035±0.004 c | 0.040±0.003 d | B |
| p12 | 2-ethenyl-3,5-dimethylpyrazine | 1535 | 0.191±0.007 a | 0.180±0.007 a | 0.183±0.001 a | 0.183±0.003 a | A |
| p13 | 2-(3-methylbutyl)pyrazine | 1577 | 0.031±0.004 a | 0.053±0.002 b | 0.057±0.005 b | 0.060±0.001 b | B |
| p14 | 2-methyl-6-(3-methylbutyl)pyrazine | 1615 | 0.065±0.001 c | 0.051±0.001 b | 0.023±0.001 a | 0.084±0.001 d | C |
| p15 | 2,5-dimethyl-3-(3-methylbutyl)pyrazine | 1650 | 0.315±0.013 a | 0.376±0.009 b | 0.415±0.037 b | 0.398±0.002 b | C |
| p16 | 1-(5-methylpyrazin-2-yl)ethanone | 1678 | N.D. a | 0.034±0.007 b | N.D. a | N.D. a | B |
| p17 | 1-(6-methylpyrazin-2-yl)ethanone | 1687 | 0.010±0.001 a | 0.016±0.001 b | 0.015±0.002 b | 0.016±0.001 b | B |
| p18 | 2-(2-phenylethyl)pyrazine | 2344 | 0.084±0.002 a | 0.086±0.004 a | 0.088±0.004 a | 0.085±0.002 a | B |
|  | ***Sulfides*** |  |  |  |  |  |  |
| s1 | (methyldisulfanyl)methane | 1075 | 0.031±0.002 a | 0.028±0.004 a | 0.041±0.011 a | 0.030±0.004 a | B |
| s2 | (methyltrisulfanyl)methane | 1375 | N.D. a | N.D. a | 0.009±0.002 b | N.D. a | B |
| s3 | phenyl(1,3-thiazol-2-yl)methanone | >2600 | 0.134±0.007 a | 0.154±0.010 a | 0.156±0.008 a | 0.150±0.002 a | A |

Table S3. cont.

|  | ***N-containing compounds*** |  |  |  |  |  |  |
| --- | --- | --- | --- | --- | --- | --- | --- |
| n3 | 3-methylbutanamide | 1913 | 0.029±0.001 b | 0.020±0.004 a | 0.026±0.002 b | 0.026±0.001 b | B |
| n4 | 2-phenylacetonitrile | 1923 | 0.034±0.002 c | 0.030±0.002 b | N.D. a | 0.035±0.002 c | A |
| n5 | 2-methylbenzonitrile | 1923 | N.D. a | N.D. a | 0.035±0.002 b | N.D. a | B |
| n6 | 3-phenylpyridine | 2231 | 0.032±0.002 a | 0.030±0.003 a | 0.031±0.002 a | 0.033±0.003 a | A |
| n7 | 2-phenylpyrimidine | 2237 | 0.059±0.005 a | 0.059±0.003 a | 0.058±0.002 a | 0.053±0.003 a | C |
| n8 | 1H-indole | 2448 | 0.052±0.006 a | 0.071±0.003 b | 0.087±0.007 c | 0.070±0.004 b | A |
| n10 | naphthalen-2-amine | >2600 | 0.094±0.008 b | 0.088±0.003 b | N.D. a | N.D. a | C |

^a^ Numbered in the order of retention indices (RI).
^b^ Retention indices (RI) were obtained using n-alkanes (C7-C26).
^c^ Mean values of relative peak area to that of internal standard ± standard deviation.
^d^ Identification of the compounds was performed as follows: C, mass spectrum was matched with manual interpretation and W9N08 (tentative identification); B, retention index and mass spectrum were consistent with those from the NIST Chemistry Webbook (tentative identification); A, retention index and mass spectrum matched with those of authentic compounds (positive identification).
^e^ There are significant differences (*p*<0.05) between samples with different lowercase letters, according to the addition of different sugars, using Duncan’s multiple range test
^f^ N.D: Not detected.

Table S4. Non-volatile metabolites identified in the samples cultivated by *B. subtilis*

| **No.^a^** | **Nonvolatile metabolites** | **Qt m/z^b^** | **Relative peak area^c^ (Mean ± SD)** | | |
| --- | --- | --- | --- | --- | --- |
|  |  |  | **BC** | **BF** | **BG** |
| ***Amino acids and its derivatives*** | | | | | |
| A1 | alanine | 116 | 10.181±0.70 b^d^ | 7.938±0.329 b | 4.749±2.306 a |
| A2 | N-methylalanine | 117 | 0.019±0.001 b | 0.021±0.003 b | 0.013±0.001 a |
| A3 | valine | 144 | 1.056±0.055 c | 0.181±0.046 b | 0.089±0.005 a |
| A4 | N-acetylglycine | 104 | 0.002±0.000 a | 0.002±0.002 a | 0.002±0.001 a |
| A5 | isoleucine | 158 | 0.571±0.064 a | 0.309±0.231 a | 0.314±0.182 a |
| A6 | proline | 142 | 3.543±0.101 c | 0.320±0.014 b | 0.171±0.018 a |
| A7 | glycine | 174 | 1.974±0.084 b | 0.691±0.093 a | 0.744±0.208 a |
| A8 | serine | 204 | 0.226±0.025 a | 0.393±0.039 b | 0.465±0.095 b |
| A9 | homoserine | 103 | 0.012±0.001 b | 0.014±0.003 ab | 0.009±0.002 a |
| A10 | threonine | 117 | 3.795±0.224 a | 5.027±0.583 b | 4.147±0.690 ab |
| A11 | allothreonine | 117 | 1.252±0.050 c | 0.780±0.151 b | 0.547±0.059 a |
| A12 | beta alanine | 174 | 0.013±0.003 b | 0.003±0.001 a | 0.002±0.001 a |
| A13 | 3-aminoisobutyric acid | 174 | 1.848±0.465 b | 0.192±0.058 a | 0.431±0.340 a |
| A14 | aspartic acid | 232 | 0.629±0.056 c | 0.241±0.050 a | 0.437±0.105 b |
| A15 | methionine | 176 | 0.724±0.262 b | 0.016±0.005 a | 0.016±0.009 a |
| A16 | glutamate | 101 | 0.051±0.001 a | 0.070±0.007 ab | 0.095±0.021 b |
| A17 | oxoproline | 156 | 1.703±0.202 b | 0.058±0.008 a | 0.790±0.652 a |
| A18 | 4-aminobutyric acid | 174 | 0.996±0.149 c | 0.691±0.066 b | 0.429±0.067 a |
| A19 | cysteine | 218 | 0.013±0.002 b | 0.002±0.002 a | trace a |

Table S4. cont.

| A20 | glutamic acid | 246 | 2.808±0.139 c | 0.643±0.150 b | 0.266±0.099 a |
| --- | --- | --- | --- | --- | --- |
| A21 | phenylalanine | 218 | 0.565±0.065 b | 0.583±0.075 b | 0.419±0.048 a |
| A22 | N-acetylaspartic acid | 158 | 0.065±0.003 b | 0.007±0.001 a | 0.011±0.004 a |
| A23 | asparagine | 116 | 0.060±0.004 a | 0.061±0.005 a | 0.057±0.023 a |
| A24 | glutamine | 156 | 71.2±15.2 b | 2.47±0.915 a | 4.35±1.17 a |
| A25 | N-acetylglutamate | 158 | 0.105±0.004 b | 0.020±0.004 a | 0.026±0.006 a |
| A26 | N-carbamoylaspartate | 147 | 0.211±0.010 a | 3.10±1.14 b | 2.89±1.58 b |
| A27 | ornithine | 142 | 179±21.7 b | 2.80±1.06 a | 5.31±2.05 a |
| A28 | citrulline | 157 | 0.036±0.025 a | 0.079±0.014 b | 0.118±0.014 c |
| A29 | O-phosphoserine | 299 | 0.414±0.575 a | 0.256±0.419 a | 0.199±0.175 a |
| A30 | methionine sulfoxide | 128 | 0.182±0.010 b | 0.042±0.004 a | 0.044±0.007 a |
| A31 | lysine | 156 | 1.82±0.140 a | 2.25±0.451 a | 2.19±0.354 a |
| A32 | histidine | 147 | 1.16±0.050 ab | 1.85±0.269 b | 0.789±0.658 a |
| A33 | tyrosine | 218 | 1.356±0.164 b | 0.252±0.040 a | 0.270±0.049 a |
| A34 | 2,6-diaminopimelic acid | 200 | 34.4±1.34 c | 5.01±1.76 a | 11.7±2.78 b |
| A35 | N-acetylornithine | 174 | 3.48±0.739 b | 0.502±0.266 a | 1.12±0.061 a |
| A36 | glucosaminic acid | 117 | 0.024±0.002 c | 0.010±0.001 a | 0.013±0.001 b |
| A37 | n-acetyl-d-hexosamine | 147 | 0.007±0.001 a | 1.56±0.292 b | 3.70±0.459 c |
| A38 | cystathionine | 128 | 0.002±0.000 a | 0.005±0.001 b | 0.005±0.001 b |
| A39 | tryptophan | 202 | 0.117±0.017 c | 0.047±0.011 a | 0.080±0.004 b |
| A40 | 5-methoxytryptamine | 174 | 0.025±0.005 a | 0.023±0.007 a | 0.039±0.006 b |
| A41 | homocystine | 128 | 0.056±0.004 a | 0.104±0.015 b | 0.172±0.035 c |

Table S4. cont.

| ***Fatty acids*** | | | | | |
| --- | --- | --- | --- | --- | --- |
| F1 | 2-hydroxyhexanoic acid | 159 | 0.002±0.000 a | 0.001±0.000 a | 0.001±0.001 a |
| F2 | caprylic acid | 117 | 0.010±0.002 a | 0.011±0.001 a | 0.014±0.001 b |
| F3 | citraconic acid | 147 | 0.276±0.023 b | 0.117±0.006 a | 0.091±0.010 a |
| F4 | pelargonic acid | 117 | 0.009±0.002 b | 0.005±0.001 a | 0.006±0.000 a |
| F5 | capric acid | 117 | 0.004±0.001 b | 0.002±0.000 a | 0.002±0.001 a |
| F6 | lauric acid | 117 | 0.045±0.001 c | 0.029±0.003 b | 0.019±0.003 a |
| F7 | myristic acid | 117 | 0.103±0.005 b | 0.066±0.002 a | 0.065±0.009 a |
| F8 | isopentadecanoic acid | 117 | 0.338±0.019 a | 0.183±0.156 a | 0.255±0.028 a |
| F9 | pentadecanoic acid | 117 | 0.040±0.003 c | 0.007±0.001 a | 0.023±0.002 b |
| F10 | isohexonic acid | 147 | 0.117±0.001 b | 0.045±0.005 a | 0.111±0.004 b |
| F11 | palmitoleic acid | 117 | 0.018±0.001 a | 0.057±0.018 b | 0.061±0.017 b |
| F12 | beta-hydroxymyristic acid | 147 | 0.274±0.007 a | 0.205±0.076 a | 0.283±0.110 a |
| F13 | palmitic acid | 117 | 3.39±0.159 a | 3.36±0.169 a | 3.07±0.234 a |
| F14 | isoheptadecanoic acid | 117 | 0.130±0.009 b | 0.057±0.012 a | 0.060±0.006 a |
| F15 | linoleic acid | 129 | 0.022±0.004 a | 0.017±0.003 a | 0.016±0.003 a |
| F16 | oleic acid | 117 | 0.023±0.006 a | 0.023±0.008 a | 0.015±0.006 a |
| F17 | stearic acid | 117 | 1.92±1.66 a | 2.95±0.185 a | 2.67±0.196 a |
| F18 | arachidic acid | 117 | 0.040±0.001 a | 0.051±0.005 a | 0.043±0.009 a |
| F19 | behenic acid | 117 | 0.005±0.001 a | 0.006±0.005 a | 0.002±0.000 a |
| F20 | lignoceric acid | 117 | 0.007±0.004 a | 0.004±0.000 a | 0.003±0.000 a |
| F21 | cerotinic acid | 117 | 0.004±0.001 a | 0.004±0.000 a | 0.004±0.001 a |

Table S4. cont.

| ***Organic acids*** | | | | | |
| --- | --- | --- | --- | --- | --- |
| O1 | pyruvic acid | 123 | 0.011±0.001 a | 0.010±0.001 a | 0.174±0.235 a |
| O2 | lactic acid | 147 | 2.60±0.641 b | 1.49±0.358 a | 0.949±0.130 a |
| O3 | 2-ketobutyric acid | 89 | trace a | trace a | trace a |
| O4 | glycolic acid | 147 | 0.031±0.014 a | 0.023±0.002 a | 0.025±0.013 a |
| O5 | maleimide | 154 | trace a | trace a | trace a |
| O6 | 2-hydroxybutanoic acid | 131 | 0.007±0.001 a | 0.037±0.004 b | 0.006±0.001 a |
| O7 | oxalic acid | 147 | 0.002±0.000 a | 0.002±0.000 a | 0.003±0.001 b |
| O8 | 3-hydroxypropionic acid | 177 | 0.001±0.0 b | trace a | trace a |
| O9 | malonic acid | 147 | 0.029±0.002 b | 0.014±0.001 b | 0.015±0.003 a |
| O10 | maleic acid | 147 | 0.655±0.048 b | 0.446±0.009 a | 0.423±0.008 a |
| O11 | nicotinic acid | 180 | 0.075±0.002 c | 0.042±0.004 b | 0.032±0.001 a |
| O12 | succinic acid | 147 | 0.362±0.018 b | 0.058±0.001 a | 0.062±0.012 a |
| O13 | 2-picolinic acid | 180 | 0.003±0.000 b | 0.002±0.000 a | 0.002±0.000 a |
| O14 | 2-deoxytetronic acid | 117 | 0.008±0.001 b | 0.003±0.001 a | 0.003±0.001 a |
| O15 | fumaric acid | 245 | 0.340±0.029 b | 0.137±0.007 a | 0.107±0.013 a |
| O16 | pyrrole-2-carboxylic acid | 240 | 0.008±0.002 b | 0.003±0.000 a | 0.002±0.000 a |
| O17 | glutaric acid | 97 | trace b | trace ab | trace a |
| O18 | hydrocinnamic acid | 104 | 0.247±0.068 b | 0.046±0.003 a | 0.044±0.003 a |
| O19 | 2-deoxytetronic acid | 189 | 0.001±0.000 b | trace a | trace a |
| O20 | malate | 147 | 0.044±0.006 b | 0.012±0.003 a | 0.016±0.005 a |
| O21 | trans-4-hydroxyproline | 158 | 0.001±0.000 b | trace a | trace a |

Table S4. cont.

| O22 | 2-hydroxyglutaric acid | 129 | 0.025±0.002 b | 0.010±0.001 a | 0.009±0.002 a |
| --- | --- | --- | --- | --- | --- |
| O23 | 4-hydroxyphenylacetic acid | 179 | trace a | trace a | trace a |
| O24 | guanidinosuccinate | 328 | 0.002±0.000 c | trace b | trace a |
| O25 | phthalic acid | 147 | 0.014±0.001 a | 0.010±0.016 a | trace a |
| O26 | shikimic acid | 204 | 0.051±0.005 b | 0.004±0.000 a | 0.006±0.003 a |
| O27 | citric acid | 147 | 0.142±0.009 a | 0.208±0.019 a | 0.174±0.115 a |
| O28 | p-hydroxylphenyllactic acid | 179 | 0.011±0.006 a | 0.009±0.001 a | 0.011±0.007 a |
| O29 | hexuronic acid | 147 | 1.04±0.007 c | 0.017±0.007 a | 0.080±0.003 b |
| O30 | galacturonic acid | 160 | 0.011±0.000 c | 0.004±0.002 b | trace a |
| O31 | pantothenic acid | 103 | 0.063±0.001 a | 0.007±0.000 a | 0.085±0.133 a |
| O32 | galactonic acid | 147 | 0.035±0.002 c | 0.009±0.001 a | 0.015±0.002 b |
| O33 | phosphogluconic acid | 204 | trace a | 0.008±0.001 b | 0.009±0.001 b |
| O34 | lactobionic acid | 204 | 0.014±0.001 c | 0.007±0.000 b | 0.005±0.001 a |

Table S4. cont.

| ***Sugar, sugar alcohols, and sugar acids*** | | | | | |
| --- | --- | --- | --- | --- | --- |
| S1 | glycerol | 147 | 467±40.1 c | 225±174 b | 16.4±12.7 a |
| S2 | glyceric acid | 147 | 3.65±0.144 b | 1.111±0.165 a | 1.06±0.423 a |
| S3 | threose | 147 | 6.14±1.10 ab | 4.95±0.867 a | 9.28±3.26 b |
| S4 | erythritol | 147 | 1691±68.3 a | 1112±199 a | 2593±604 b |
| S5 | threonic acid | 147 | 0.032±0.044 a | 0.018±0.012 a | 0.020±0.001 a |
| S6 | isothreonic acid | 147 | 0.421±0.074 b | 0.053±0.004 a | 0.068±0.003 a |
| S7 | tartaric acid | 147 | 1.06±0.342 b | 0.092±0.029 a | 0.029±0.024 a |
| S8 | arabinose | 103 | 21.6±3.78 ab | 8.08±7.33 a | 25.4±8.47 b |
| S9 | ribose | 118 | 3.49±0.320 a | 4.25±2.731 a | 20.6±8.50 b |
| S10 | lyxose | 103 | 24.7±0.976 a | 131±23.8 a | 373±155 b |
| S11 | xylulose | 147 | 31.946±7.70 a | 154.164±67.429 b | 505.451±32.527 c |
| S12 | isoribose | 103 | 0.106±0.054 a | 2.36±0.562 a | 7.55±3.61 b |
| S13 | arabitol | 147 | 4.62±0.964 a | 19.6±7.31 ab | 20.5±10.7 b |
| S14 | 6-deoxyglucose | 117 | 24.107±1.134 c | 4.051±2.221 a | 14.197±7.370 b |
| S15 | ribitol | 147 | 51.1±2.43 b | 17.3±6.36 a | 20.8±6.47 a |
| S16 | ribonic acid | 117 | 0.005±0.000 b | 0.002±0.002 a | 0.004±0.000 b |
| S17 | 3-phosphoglycerate | 147 | 0.212±0.011 a | 1.661±0.120 b | 2.249±0.541 b |
| S18 | 1,5-anhydroglucitol | 133 | 51.2±3.46 c | 6.44±1.37 a | 21.9±5.34 b |
| S19 | fructose | 103 | 52.2±5.38 a | 126±76.3 a | 338±74.3 b |
| S20 | galactose | 147 | 64.0±11.7 b | 15.6±3.73 a | 104±23.4 c |
| S21 | glucose | 147 | 82.1±5.27 a | 26.6±6.03 a | 168±58.9 b |

Table S4. cont.

| S22 | Mannitol | 117 | 415±23.8 b | 205±72.4 a | 442±131 b |
| --- | --- | --- | --- | --- | --- |
| S23 | sorbitol | 147 | 359±11.0 c | 9.47±2.20 a | 27.4±7.26 b |
| S24 | galactitol | 147 | 353±16.9 b | 10.1±2.78 a | 24.0±8.22 a |
| S25 | mannonic acid | 204 | 0.001±0.000 a | 0.008±0.001 b | 0.065±0.002 c |
| S26 | gluconic acid | 147 | 0.019±0.000 a | 0.071±0.007 b | 0.636±0.019 c |
| S27 | hexitol | 318 | 174±12.6 b | 3.35±0.685 a | 6.34±2.82 a |
| S28 | glucoheptulose | 103 | 0.308±0.106 a | 4.56±1.36 a | 54.8±14.1 b |
| S29 | sucrose | 117 | 39.8±2.79 a | 25.8±9.09 a | 30.5±11.2 a |
| S30 | lactulose | 147 | 1.36±0.151 a | 1.87±1.58 a | 1.72±1.18 a |
| S31 | cellobiose | 100 | 2.08±0.347 a | 3.36±1.102 a | 12.9±4.03 b |
| S32 | lactose | 160 | 34.5±5.36 b | 4.83±1.19 a | 6.98±3.71 a |
| S33 | trehalose | 147 | 3.19±0.308 a | 3.92±1.05 a | 8.13±1.80 b |
| S34 | maltose | 361 | 1.0±0.162 b | 0.224±0.052 a | 0.451±0.152 a |
| S35 | beta-gentiobiose | 204 | 6.57±0.395 b | 0.657±0.082 a | 3.51±2.71 ab |
| S36 | maltitol | 204 | 30.4±1.74 b | 4.93±1.13 a | 4.25±2.04 a |
| S37 | isomaltose | 147 | 7.49±5.84 a | 0.970±0.269 a | 2.88±0.748 a |
| S38 | melibiose | 204 | 6.61±0.148 c | 1.86±0.343 b | 0.977±0.214 a |
| S39 | palatinitol | 147 | 0.036±0.008 b | 1.470±0.226 b | 1.608±0.852 a |
| S40 | galactinol | 204 | 37.1±2.34 b | 9.83±5.30 a | 13.9±4.73 a |
| S41 | raffinose | 217 | 188±18.4 b | 0.664±0.177 a | 1.01±0.215 a |
| S42 | 1-kestose | 361 | 177±18.8 b | 0.770±0.197 a | 0.968±0.205 a |
| S43 | melezitose | 361 | 7.67±0.735 b | 0.026±0.006 a | 0.035±0.030 a |
| S44 | maltotriitol | 129 | 85.7±11.4 b | 8.03±6.97 a | 20.2±7.43 a |
| S45 | trisaccharide | 204 | 22.5±13.1 b | 0.098±0.069 a | 0.285±0.377 a |

Table S4. cont.

| ***Others*** | | | | | |
| --- | --- | --- | --- | --- | --- |
| Z1 | butane-2,3-diol | 117 | trace a | trace a | 0.001±0.002 a |
| Z2 | propane-1,3-diol | 147 | 0.001±0.001 a | 0.153±0.262 a | 0.307±0.406 a |
| Z3 | phenol | 151 | 0.011±0.004 a | 0.006±0.001 a | 0.019±0.023 a |
| Z4 | isobutene glycol | 147 | 0.031±0.014 a | 0.025±0.003 a | 0.024±0.014 a |
| Z5 | enolpyruvate | 147 | 0.007±0.001 a | 0.108±0.016 b | 0.135±0.019 b |
| Z6 | octanol | 187 | 0.001±0.000 a | trace a | 0.002±0.001 a |
| Z7 | hydroxylamine | 133 | 0.003±0.000 a | 0.003±0.000 a | 0.004±0.001 b |
| Z8 | butyrolactam | 142 | 0.010±0.001 b | 0.005±0.000 a | 0.006±0.000 a |
| Z9 | sulfuric acid | 95 | 0.005±0.0 a | 0.005±0.000 a | 0.005±0.000 a |
| Z10 | methanolphosphate | 241 | 0.019±0.001 c | 0.012±0.001 b | 0.010±0.001 a |
| Z11 | urea | 147 | 0.001±0.001 a | trace a | 0.004±0.007 a |
| Z12 | pyrophosphate | 110 | 0.039±0.005 c | 0.026±0.001 b | 0.010±0.003 a |
| Z13 | ethanolamine | 174 | 0.026±0.001 b | 0.013±0.000 a | 0.013±0.002 a |
| Z14 | phosphate | 299 | 1.63±0.062 a | 1.51±0.364 a | 1.66±0.124 a |
| Z15 | uracil | 184 | 0.014±0.001 a | 0.027±0.002 ab | 0.057±0.026 b |
| Z16 | 4-methyl-5-thiazoleethanol | 102 | trace a | trace a | trace a |
| Z17 | thymine | 255 | 0.007±0.000 c | 0.004±0.001 b | 0.002±0.000 a |
| Z18 | nicotinamide | 179 | 0.165±0.013 a | 0.334±0.004 b | 0.314±0.019 b |
| Z19 | creatinine | 115 | 0.010±0.001 c | 0.005±0.001 b | 0.002±0.001 a |
| Z20 | dithiothreitol | 116 | 0.743±0.016 b | 0.288±0.036 a | 0.337±0.101 a |
| Z21 | levoglucosan | 204 | 0.003±0.000 a | 0.004±0.001 b | 0.003±0.000 ab |

Table S4. cont.

| Z22 | beta-glycerolphosphate | 243 | 0.010±0.000 b | 0.003±0.000 a | 0.003±0.000 a |
| --- | --- | --- | --- | --- | --- |
| Z23 | UDP-glucuronic acid | 217 | 0.004±0.000 a | 0.004±0.000 a | 0.004±0.001 a |
| Z24 | putrescine | 174 | 0.004±0.000 a | 0.005±0.000 ab | 0.008±0.003 b |
| Z25 | glycerol-alpha-phosphate | 299 | 0.230±0.006 b | 0.077±0.006 a | 0.067±0.006 a |
| Z26 | diglycerol | 147 | 0.137±0.107 a | 0.051±0.039 a | 0.061±0.006 a |
| Z27 | N-acetylputrescine | 174 | 0.018±0.004 a | 0.109±0.017 c | 0.060±0.019 b |
| Z28 | glucose-1-phosphate | 217 | 0.004±0.000 a | 0.009±0.001 b | 0.012±0.002 c |
| Z29 | phosphoethanolamine | 100 | 0.027±0.002 b | 0.025±0.001 b | 0.017±0.001 a |
| Z30 | thymidine-5-phosphate | 211 | 0.003±0.000 a | 0.004±0.002 a | 0.004±0.000 a |
| Z31 | methylhexose | 174 | 0.023±0.026 a | 0.017±0.002 a | 0.025±0.001 a |
| Z32 | hypoxanthine | 117 | 0.085±0.002 c | 0.027±0.001 a | 0.039±0.001 b |
| Z33 | pinitol | 147 | 0.657±0.018 b | 0.072±0.003 a | 0.060±0.002 a |
| Z34 | UDP-N-acetylglucosamine | 147 | 0.657±0.018 b | 0.072±0.003 a | 0.060±0.002 a |
| Z35 | UDP GlcNAc | 147 | 0.044±0.001 b | 0.006±0.001 a | 0.004±0.000 a |
| Z36 | alanine-alanine | 174 | 0.822±0.022 b | 0.720±0.032 a | 0.736±0.062 a |
| Z37 | allantoin | 188 | trace a | 0.001±0.000 a | 0.007±0.002 b |
| Z38 | adenine | 264 | 0.159±0.012 c | 0.036±0.003 b | 0.014±0.001 a |
| Z39 | gluconic acid lactone | 129 | 0.003±0.000 a | 0.010±0.003 a | 0.057±0.013 b |
| Z40 | 1,2-anhydro-myo-inositol | 204 | 0.044±0.005 b | 0.003±0.001 a | 0.005±0.003 a |
| Z41 | ononitol | 147 | 0.137±0.006 b | 0.008±0.002 a | 0.010±0.004 a |
| Z42 | glycyl-proline | 174 | 0.007±0.000 b | 0.003±0.000 a | 0.004±0.001 a |
| Z43 | xanthine | 353 | 0.013±0.001 b | 0.003±0.003 a | trace a |

Table S4. cont.

| Z44 | conduritol-beta-expoxide | 147 | 1.30±0.003 b | 0.210±0.076 a | 0.258±0.157 a |
| --- | --- | --- | --- | --- | --- |
| Z45 | myo-inositol | 147 | 0.274±0.095 b | 0.064±0.013 a | 0.033±0.005 a |
| Z46 | ribose-5-phosphate | 315 | 0.002±0.001 a | 0.006±0.001 b | 0.009±0.002 c |
| Z47 | ribulose-5-phosphate | 147 | 0.008±0.008 a | 0.006±0.009 a | 0.279±0.482 a |
| Z48 | guanine | 169 | 0.001±0.000 a | 0.004±0.001 b | 0.010±0.001 c |
| Z49 | octadecanol | 327 | 0.001±0.000 a | 0.002±0.001 a | 0.002±0.000 a |
| Z50 | noradrenaline | 174 | 0.001±0.000 a | 0.003±0.000 b | 0.004±0.001 c |
| Z51 | bisphosphoglycerol | 299 | trace a | 0.006±0.003 b | 0.002±0.001 a |
| Z52 | spermidine | 174 | 0.011±0.002 a | 0.004±0.000 a | 0.009±0.009 a |
| Z53 | fructose-1-phosphate | 103 | 0.001±0.001 a | 0.027±0.015 b | 0.027±0.014 b |
| Z54 | fructose-6-phosphate | 315 | trace a | 0.003±0.001 a | 0.006±0.002 b |
| Z55 | glycerol-3-galactoside | 204 | 0.010±0.001 b | trace a | trace a |
| Z56 | glucose-6-phosphate | 147 | 0.057±0.004 a | 0.048±0.001 a | 0.10±0.022 b |
| Z57 | mannose-6-phosphate | 204 | 0.011±0.003 a | 0.011±0.000 a | 0.018±0.002 b |
| Z58 | pyridoxal-5-phosphate | 219 | 0.001±0.000 b | trace ab | trace a |
| Z59 | galactose-6-phosphate | 204 | 0.003±0.000 a | 0.003±0.002 a | 0.005±0.001 a |
| Z60 | tetracosane | 85 | 0.006±0.000 b | 0.004±0.001 a | 0.005±0.000 a |
| Z61 | cytidine-5-monophosphate | 142 | 0.037±0.003 a | 0.089±0.011 b | 0.094±0.003 b |
| Z62 | oleamide | 201 | 0.003±0.000 a | 0.002±0.000 a | 0.003±0.001 a |
| Z63 | n-acetyl-5-hydroxytryptamine | 129 | 0.015±0.001 b | 0.010±0.002 a | 0.011±0.003 ab |
| Z64 | phosphohexonic acid | 147 | 0.041±0.033 a | 0.034±0.016 a | 0.047±0.014 a |
| Z65 | uridine | 217 | 0.123±0.065 a | 0.047±0.018 a | 0.078±0.026 a |

Table S4. cont.

| Z66 | serotonin | 174 | 0.003±0.000 a | 0.003±0.000 ab | 0.003±0.000 b |
| --- | --- | --- | --- | --- | --- |
| Z67 | 2-monopalmitin | 129 | 0.008±0.001 a | 0.024±0.030 a | 0.132±0.038 b |
| Z68 | inosine | 217 | 0.004±0.001 b | trace a | trace a |
| Z69 | 1-monopalmitin | 147 | 0.011±0.001 a | 0.016±0.001 b | 0.016±0.004 b |
| Z70 | adenosine | 236 | 0.040±0.010 b | 0.024±0.001 a | 0.014±0.002 a |
| Z71 | xanthosine | 147 | 0.024±0.003 b | 0.008±0.001 a | 0.007±0.007 a |
| Z72 | guanosine | 204 | 0.019±0.002 c | 0.003±0.000 a | 0.006±0.001 b |
| Z73 | 1-monostearin | 147 | 0.001±0.000 a | 0.004±0.000 c | 0.003±0.000 b |
| Z74 | 5'-deoxy-5'-methylthioadenosine | 236 | 0.010±0.001 b | 0.011±0.001 b | 0.008±0.001 a |
| Z75 | uridine-5'-monophosphate | 169 | 0.003±0.001 b | trace a | trace a |
| Z76 | 3'-adenylic acid | 315 | trace a | trace a | trace a |
| Z77 | adenosine-5-monophosphate | 169 | 0.261±0.063 a | 0.50±0.158 b | 0.214±0.105 a |
| Z78 | alpha-tocopherol | 237 | trace a | trace a | trace a |
| Z79 | cholesterol | 129 | 0.012±0.001 b | trace a | trace a |
| Z80 | beta-sitosterol | 119 | 0.143±0.010 c | 0.059±0.006 b | 0.036±0.003 a |

^a^ Numbered in the order of retention times (RI).
^b^ Quantitative m/z.
^c^ Mean values of relative peak area to that of internal standard ± standard deviation.
^d^ There are significant differences (*p*<0.05) between samples with different lowercase letters using Duncan’s multiple comparison test

**
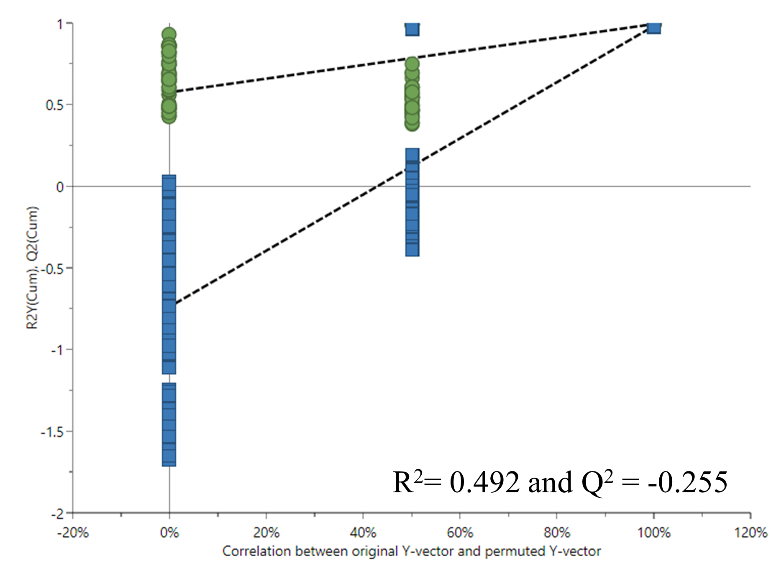
**

**Figure S1. Results of the permutation test (Figure 3)**
